# (2,2′-Bipyridine)(2-formyl-6-methoxy­phenolato)nickel(II) perchlorate

**DOI:** 10.1107/S1600536808040385

**Published:** 2008-12-06

**Authors:** Cui-Juan Wang, Ping-Di Ren, Wei-Ping Wu, Zhi-Bin Zhang

**Affiliations:** a Department of Chemistry and Chemical Engineering, School of Bioengineering, Southwest JiaoTong University, Chengdu, Sichuan 610031, People’s Republic of China; b Department of Chemistry, College of Chemistry and Pharmaceutical Engineering, Sichuan University of Science and Engineering, Zigong, Sichuan 643000, People’s Republic of China

## Abstract

In the title compound, [Ni(C_8_H_7_O_3_)(C_10_H_8_N_2_)]ClO_4_, the Ni^II^ atom is in a slightly distorted square-planar coordination by two N atoms from the 2,2′-bipyridine (bipy) ligand and two O atoms from the deprotonated 2-formyl-6-methoxy­phenolate (mbd) ligand. The bipy ligand is nearly coplanar with the Ni^II^ square plane, the Ni atom being only 0.042 (2) Å from the mean plane, whereas the benzaldehyde plane is folded with respect to the square plane, making a dihedral angle of 19.17 (8)°. One of the O atoms of the perchlorate anion is involved in a weak inter­action with the Ni atom, with an Ni—O distance of 2.5732 (18) Å. The packing is stabilized by weak C—H⋯O inter­actions.

## Related literature

For general background, see: Alizadeh *et al.* (1999 ▶); Hamblin *et al.* (2002 ▶); Minuti *et al.* (1999 ▶). For a related structure, see: Liu *et al.* (2008 ▶).
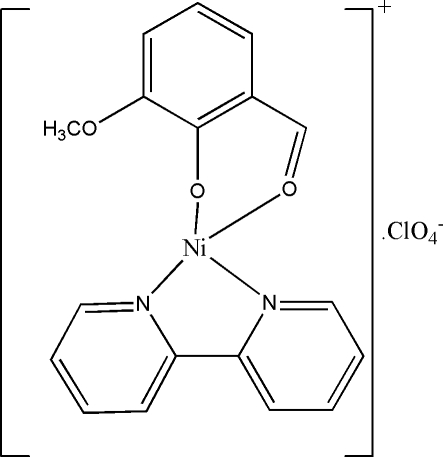


## Experimental

### 

#### Crystal data

[Ni(C_8_H_7_O_3_)(C_10_H_8_N_2_)]ClO_4_
*M* *_r_* = 465.48
Triclinic, 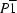

*a* = 8.460 (1) Å
*b* = 9.580 (1) Å
*c* = 11.956 (2) Å
α = 84.45 (1)°
β = 80.05 (1)°
γ = 80.25 (1)°
*V* = 938.4 (2) Å^3^
*Z* = 2
Mo *K*α radiation
μ = 1.22 mm^−1^
*T* = 298 (2) K
0.32 × 0.26 × 0.19 mm

#### Data collection

Bruker APEXII area-detector diffractometer
Absorption correction: multi-scan (*SADABS*; Bruker, 2004 ▶) *T*_min_ = 0.696, *T*_max_ = 0.801
3644 measured reflections
3381 independent reflections
2838 reflections with *I* > 2σ(*I*)
*R*_int_ = 0.009

#### Refinement

*R*[*F* ^2^ > 2σ(*F* ^2^)] = 0.029
*wR*(*F* ^2^) = 0.077
*S* = 1.04
3381 reflections
263 parameters
H-atom parameters constrained
Δρ_max_ = 0.25 e Å^−3^
Δρ_min_ = −0.50 e Å^−3^

### 

Data collection: *APEX2* (Bruker, 2004 ▶); cell refinement: *APEX2*; data reduction: *APEX2*; program(s) used to solve structure: *SHELXS97* (Sheldrick, 2008 ▶); program(s) used to refine structure: *SHELXL97* (Sheldrick, 2008 ▶); molecular graphics: *ORTEPIII* (Burnett & Johnson, 1996 ▶) and *ORTEP-3 for Windows* (Farrugia, 1997 ▶); software used to prepare material for publication: *SHELXL97*.

## Supplementary Material

Crystal structure: contains datablocks I, global. DOI: 10.1107/S1600536808040385/dn2410sup1.cif

Structure factors: contains datablocks I. DOI: 10.1107/S1600536808040385/dn2410Isup2.hkl

Additional supplementary materials:  crystallographic information; 3D view; checkCIF report

## Figures and Tables

**Table 1:** Hydrogen-bond geometry (Å, °)

*D*—H⋯*A*	*D*—H	H⋯*A*	*D*⋯*A*	*D*—H⋯*A*
C4—H4⋯O7^i^	0.93	2.58	3.461 (4)	159
C12—H12⋯O5^ii^	0.93	2.57	3.499 (3)	177
C15—H15⋯O5^ii^	0.93	2.59	3.517 (3)	171

Symmetry codes: (i) 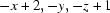; (ii) 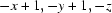.

**Fig. 1.**
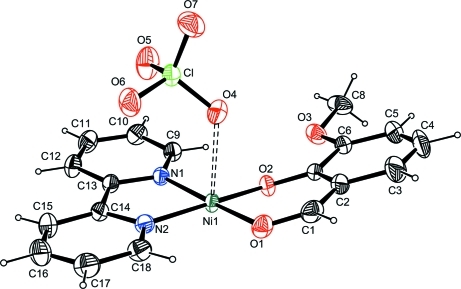
Molecular view of compound (I) with the atom-labeling scheme. Displacement ellipsoids are drawn at the 30% probability level. H atoms are represented as small spheres of arbitrary radii.

## supplementary crystallographic information

### Comment

2,2'-Bipyridyl ligand is bidentate chelating ligand, which can commonly act as terminal ligands, and may also provide molecular interaction sites for molecular recognition or assembly (Minuti *et al.*, 1999; Hamblin *et al.*, 2002). On the other hand, aldehyde ligands have significant importance in chemistry, specially in the development of its complexes, because this type ligands are potentially capable of forming stable complexes with metal ions (Alizadeh *et al.*, 1999). Herein we report the synthesis and characterization of the title complex with mixed co-ligand.

The Ni^II^ atom in the title complex, has a square coordination formed by two N atoms from Bipy ligand and two O atoms from protonated mbd ligand (Fig. 1). One of the O atom of the perchlorate anion is in weak interaction with the Ni atom with a Ni—O distance of 2.5732 (18) Å. The average Ni—N bond length of 1.98 Å is close to the values observed in related complexes (Liu *et al.*, 2008). The occurrence of C—H···O hydrogen bonding stabilizes the packing (Table 1).

### Experimental

Bipy (0.023 g, 0.12 mmol), Ni(ClO_4_)_2_ (0.028 g, 0.13 mmol) and Hmbd (0.020 g, 0.16 mmol), were added in a solvent of ethanol, the mixture was stirring were required. The resultant solution was kept at room temperature for three weeks yielding green crystals of (I)

### Refinement

All H atoms attached were fixed geometrically and treated as riding on their parent C atoms with C—H = 0.96 Å (methyl) or 0.93 Å (aromatic) with U_iso_(H) = 1.2U_eq_(aromatic) or U_iso_(H) = 1.5U_eq_(methyl).

### Crystal data

**Table:**

[Ni(C_8_H_7_O_3_)(C_10_H_8_N_2_)]ClO_4_	*Z* = 2
*M**_r_* = 465.48	*F*(000) = 476
Triclinic, *P*1	*D*_x_ = 1.647 Mg m^−^^3^
Hall symbol: -P 1	Mo *K*α radiation, λ = 0.71073 Å
*a* = 8.460 (1) Å	Cell parameters from 3381 reflections
*b* = 9.580 (1) Å	θ = 1.7–25.2°
*c* = 11.956 (2) Å	µ = 1.22 mm^−^^1^
α = 84.45 (1)°	*T* = 298 K
β = 80.05 (1)°	Block, green
γ = 80.25 (1)°	0.32 × 0.26 × 0.19 mm
*V* = 938.4 (2) Å^3^	

### Data collection

**Table:**

Bruker APEXII area-detector diffractometer	3381 independent reflections
Radiation source: fine-focus sealed tube	2838 reflections with *I* > 2σ(*I*)
graphite	*R*_int_ = 0.009
φ and ω scans	θ_max_ = 25.2°, θ_min_ = 1.7°
Absorption correction: multi-scan (*SADABS*; Bruker, 2004)	*h* = 0→10
*T*_min_ = 0.696, *T*_max_ = 0.801	*k* = −11→11
3644 measured reflections	*l* = −14→14

### Refinement

**Table:**

Refinement on *F*^2^	Primary atom site location: structure-invariant direct methods
Least-squares matrix: full	Secondary atom site location: difference Fourier map
*R*[*F*^2^ > 2σ(*F*^2^)] = 0.029	Hydrogen site location: inferred from neighbouring sites
*wR*(*F*^2^) = 0.077	H-atom parameters constrained
*S* = 1.04	*w* = 1/[σ^2^(*F*_o_^2^) + (0.049*P*)^2^] where *P* = (*F*_o_^2^ + 2*F*_c_^2^)/3
3381 reflections	(Δ/σ)_max_ = 0.001
263 parameters	Δρ_max_ = 0.25 e Å^−^^3^
0 restraints	Δρ_min_ = −0.49 e Å^−^^3^

### Special details

**Table:**

Geometry. All esds (except the esd in the dihedral angle between two l.s. planes) are estimated using the full covariance matrix. The cell esds are taken into account individually in the estimation of esds in distances, angles and torsion angles; correlations between esds in cell parameters are only used when they are defined by crystal symmetry. An approximate (isotropic) treatment of cell esds is used for estimating esds involving l.s. planes.
Refinement. Refinement of *F*^2^ against ALL reflections. The weighted *R*-factor *wR* and goodness of fit *S* are based on *F*^2^, conventional *R*-factors *R* are based on *F*, with *F* set to zero for negative *F*^2^. The threshold expression of *F*^2^ > σ(*F*^2^) is used only for calculating *R*-factors(gt) *etc*. and is not relevant to the choice of reflections for refinement. *R*-factors based on *F*^2^ are statistically about twice as large as those based on *F*, and *R*- factors based on ALL data will be even larger.

### Fractional atomic coordinates and isotropic or equivalent isotropic displacement parameters (Å^2^)

**Table:**

	*x*	*y*	*z*	*U*_iso_*/*U*_eq_	
Ni1	0.62596 (3)	0.46273 (3)	0.37323 (2)	0.03207 (11)	
O1	0.8230 (2)	0.46228 (19)	0.43851 (14)	0.0491 (4)	
O2	0.54224 (18)	0.33979 (16)	0.49436 (13)	0.0397 (4)	
O3	0.4083 (2)	0.12543 (17)	0.59302 (15)	0.0531 (5)	
N1	0.4407 (2)	0.45896 (19)	0.29274 (15)	0.0356 (4)	
N2	0.6801 (2)	0.6028 (2)	0.24547 (15)	0.0384 (4)	
C1	0.8713 (3)	0.3669 (3)	0.5099 (2)	0.0539 (7)	
H1	0.9750	0.3677	0.5258	0.065*	
C2	0.7895 (3)	0.2585 (3)	0.5693 (2)	0.0490 (6)	
C3	0.8710 (4)	0.1578 (3)	0.6437 (3)	0.0797 (10)	
H3	0.9760	0.1654	0.6530	0.096*	
C4	0.7985 (5)	0.0514 (3)	0.7010 (3)	0.0935 (13)	
H4	0.8532	−0.0131	0.7499	0.112*	
C5	0.6424 (4)	0.0376 (3)	0.6871 (2)	0.0711 (9)	
H5	0.5940	−0.0367	0.7267	0.085*	
C6	0.5578 (3)	0.1319 (2)	0.6157 (2)	0.0462 (6)	
C7	0.6294 (3)	0.2479 (2)	0.55657 (18)	0.0390 (5)	
C8	0.3359 (4)	0.0033 (3)	0.6415 (2)	0.0662 (8)	
H8A	0.4119	−0.0814	0.6251	0.099*	
H8B	0.2399	0.0015	0.6094	0.099*	
H8C	0.3075	0.0084	0.7225	0.099*	
C9	0.3265 (3)	0.3750 (3)	0.3212 (2)	0.0416 (5)	
H9	0.3278	0.3148	0.3870	0.050*	
C10	0.2074 (3)	0.3751 (3)	0.2563 (2)	0.0503 (6)	
H10	0.1300	0.3151	0.2774	0.060*	
C11	0.2044 (3)	0.4647 (3)	0.1599 (2)	0.0544 (7)	
H11	0.1239	0.4668	0.1154	0.065*	
C12	0.3211 (3)	0.5518 (3)	0.1291 (2)	0.0512 (6)	
H12	0.3208	0.6129	0.0637	0.061*	
C13	0.4388 (3)	0.5464 (2)	0.19728 (18)	0.0380 (5)	
C14	0.5734 (3)	0.6303 (2)	0.17170 (18)	0.0393 (5)	
C15	0.5939 (4)	0.7291 (3)	0.0798 (2)	0.0557 (7)	
H15	0.5181	0.7493	0.0304	0.067*	
C16	0.7279 (4)	0.7967 (3)	0.0627 (2)	0.0634 (8)	
H16	0.7435	0.8629	0.0011	0.076*	
C17	0.8374 (4)	0.7669 (3)	0.1357 (2)	0.0594 (7)	
H17	0.9291	0.8113	0.1244	0.071*	
C18	0.8102 (3)	0.6699 (3)	0.2267 (2)	0.0484 (6)	
H18	0.8848	0.6500	0.2770	0.058*	
Cl	0.82646 (7)	0.24175 (6)	0.14548 (5)	0.04118 (15)	
O4	0.8132 (2)	0.2621 (2)	0.26486 (14)	0.0597 (5)	
O5	0.6696 (2)	0.2285 (2)	0.12270 (17)	0.0753 (6)	
O6	0.8814 (3)	0.3620 (2)	0.07874 (16)	0.0652 (5)	
O7	0.9370 (3)	0.1160 (2)	0.11921 (17)	0.0757 (6)	

### Atomic displacement parameters (Å^2^)

**Table:**

	*U*^11^	*U*^22^	*U*^33^	*U*^12^	*U*^13^	*U*^23^
Ni1	0.03248 (17)	0.03601 (17)	0.03033 (16)	−0.01101 (12)	−0.01119 (11)	0.00581 (11)
O1	0.0452 (10)	0.0571 (11)	0.0506 (10)	−0.0154 (8)	−0.0172 (8)	−0.0019 (9)
O2	0.0396 (9)	0.0392 (9)	0.0400 (9)	−0.0051 (7)	−0.0130 (7)	0.0092 (7)
O3	0.0633 (12)	0.0395 (10)	0.0524 (10)	−0.0135 (9)	0.0016 (9)	0.0073 (8)
N1	0.0376 (10)	0.0365 (10)	0.0333 (9)	−0.0057 (8)	−0.0090 (8)	0.0003 (8)
N2	0.0423 (11)	0.0382 (11)	0.0351 (10)	−0.0104 (9)	−0.0028 (8)	−0.0029 (8)
C1	0.0450 (15)	0.0599 (17)	0.0627 (17)	−0.0026 (13)	−0.0254 (13)	−0.0138 (14)
C2	0.0554 (16)	0.0417 (14)	0.0545 (15)	0.0006 (12)	−0.0293 (13)	−0.0036 (12)
C3	0.087 (2)	0.059 (2)	0.104 (3)	0.0030 (18)	−0.065 (2)	0.0011 (19)
C4	0.133 (3)	0.053 (2)	0.109 (3)	−0.004 (2)	−0.084 (3)	0.0223 (19)
C5	0.116 (3)	0.0403 (16)	0.0610 (18)	−0.0093 (17)	−0.0372 (18)	0.0122 (13)
C6	0.0663 (17)	0.0351 (13)	0.0367 (12)	−0.0047 (12)	−0.0126 (12)	0.0014 (10)
C7	0.0525 (14)	0.0325 (12)	0.0317 (11)	0.0029 (11)	−0.0144 (10)	−0.0039 (9)
C8	0.090 (2)	0.0397 (15)	0.0617 (18)	−0.0221 (15)	0.0168 (16)	0.0003 (13)
C9	0.0405 (13)	0.0436 (14)	0.0427 (13)	−0.0110 (11)	−0.0101 (10)	0.0012 (11)
C10	0.0427 (14)	0.0594 (17)	0.0530 (15)	−0.0150 (13)	−0.0107 (12)	−0.0059 (13)
C11	0.0469 (15)	0.0707 (19)	0.0503 (15)	−0.0073 (14)	−0.0226 (12)	−0.0046 (14)
C12	0.0531 (15)	0.0616 (17)	0.0414 (14)	−0.0082 (13)	−0.0201 (12)	0.0045 (12)
C13	0.0421 (13)	0.0389 (12)	0.0320 (11)	−0.0016 (10)	−0.0085 (10)	−0.0009 (10)
C14	0.0454 (14)	0.0381 (13)	0.0330 (11)	−0.0040 (11)	−0.0050 (10)	−0.0019 (10)
C15	0.0692 (18)	0.0561 (17)	0.0394 (14)	−0.0127 (14)	−0.0074 (13)	0.0109 (12)
C16	0.082 (2)	0.0588 (18)	0.0454 (15)	−0.0230 (16)	0.0032 (15)	0.0125 (13)
C17	0.0655 (19)	0.0593 (18)	0.0523 (16)	−0.0256 (15)	0.0086 (14)	−0.0028 (14)
C18	0.0479 (14)	0.0509 (15)	0.0484 (14)	−0.0199 (12)	−0.0002 (11)	−0.0046 (12)
Cl	0.0437 (3)	0.0428 (3)	0.0378 (3)	−0.0086 (3)	−0.0106 (2)	0.0041 (2)
O4	0.0798 (13)	0.0620 (12)	0.0386 (9)	−0.0123 (10)	−0.0148 (9)	0.0014 (9)
O5	0.0592 (12)	0.1022 (17)	0.0741 (14)	−0.0325 (12)	−0.0310 (10)	0.0178 (12)
O6	0.0829 (14)	0.0616 (12)	0.0546 (11)	−0.0305 (11)	−0.0136 (10)	0.0177 (9)
O7	0.0816 (15)	0.0588 (13)	0.0725 (14)	0.0163 (11)	0.0001 (11)	−0.0044 (11)

### Geometric parameters (Å, °)

**Table:**

Ni1—O2	1.8932 (15)	C8—H8B	0.9600
Ni1—O1	1.9580 (16)	C8—H8C	0.9600
Ni1—N2	1.9783 (19)	C9—C10	1.374 (3)
Ni1—N1	1.9828 (18)	C9—H9	0.9300
O1—C1	1.255 (3)	C10—C11	1.370 (3)
O2—C7	1.310 (3)	C10—H10	0.9300
O3—C6	1.351 (3)	C11—C12	1.379 (4)
O3—C8	1.439 (3)	C11—H11	0.9300
N1—C9	1.339 (3)	C12—C13	1.384 (3)
N1—C13	1.349 (3)	C12—H12	0.9300
N2—C18	1.343 (3)	C13—C14	1.476 (3)
N2—C14	1.346 (3)	C14—C15	1.386 (3)
C1—C2	1.412 (4)	C15—C16	1.374 (4)
C1—H1	0.9300	C15—H15	0.9300
C2—C7	1.410 (3)	C16—C17	1.359 (4)
C2—C3	1.421 (4)	C16—H16	0.9300
C3—C4	1.348 (5)	C17—C18	1.374 (3)
C3—H3	0.9300	C17—H17	0.9300
C4—C5	1.388 (5)	C18—H18	0.9300
C4—H4	0.9300	Cl—O7	1.4194 (19)
C5—C6	1.378 (4)	Cl—O5	1.4284 (19)
C5—H5	0.9300	Cl—O6	1.4336 (18)
C6—C7	1.425 (3)	Cl—O4	1.4418 (17)
C8—H8A	0.9600		
			
O2—Ni1—O1	92.24 (7)	O3—C8—H8C	109.5
O2—Ni1—N2	171.66 (7)	H8A—C8—H8C	109.5
O1—Ni1—N2	94.93 (7)	H8B—C8—H8C	109.5
O2—Ni1—N1	91.43 (7)	N1—C9—C10	122.1 (2)
O1—Ni1—N1	174.35 (7)	N1—C9—H9	119.0
N2—Ni1—N1	81.75 (8)	C10—C9—H9	119.0
C1—O1—Ni1	122.66 (17)	C11—C10—C9	119.0 (2)
C7—O2—Ni1	125.39 (15)	C11—C10—H10	120.5
C6—O3—C8	116.8 (2)	C9—C10—H10	120.5
C9—N1—C13	119.0 (2)	C10—C11—C12	119.7 (2)
C9—N1—Ni1	126.47 (15)	C10—C11—H11	120.1
C13—N1—Ni1	114.47 (15)	C12—C11—H11	120.1
C18—N2—C14	118.7 (2)	C11—C12—C13	118.7 (2)
C18—N2—Ni1	126.43 (17)	C11—C12—H12	120.7
C14—N2—Ni1	114.84 (15)	C13—C12—H12	120.7
O1—C1—C2	128.7 (2)	N1—C13—C12	121.5 (2)
O1—C1—H1	115.7	N1—C13—C14	114.52 (19)
C2—C1—H1	115.7	C12—C13—C14	124.0 (2)
C7—C2—C1	121.7 (2)	N2—C14—C15	121.0 (2)
C7—C2—C3	119.4 (3)	N2—C14—C13	114.38 (19)
C1—C2—C3	119.0 (3)	C15—C14—C13	124.6 (2)
C4—C3—C2	121.0 (3)	C16—C15—C14	119.1 (3)
C4—C3—H3	119.5	C16—C15—H15	120.4
C2—C3—H3	119.5	C14—C15—H15	120.4
C3—C4—C5	120.2 (3)	C17—C16—C15	119.9 (3)
C3—C4—H4	119.9	C17—C16—H16	120.0
C5—C4—H4	119.9	C15—C16—H16	120.0
C6—C5—C4	121.3 (3)	C16—C17—C18	118.8 (3)
C6—C5—H5	119.4	C16—C17—H17	120.6
C4—C5—H5	119.4	C18—C17—H17	120.6
O3—C6—C5	126.1 (3)	N2—C18—C17	122.4 (3)
O3—C6—C7	114.1 (2)	N2—C18—H18	118.8
C5—C6—C7	119.8 (3)	C17—C18—H18	118.8
O2—C7—C2	123.6 (2)	O7—Cl—O5	109.87 (14)
O2—C7—C6	118.2 (2)	O7—Cl—O6	110.45 (13)
C2—C7—C6	118.3 (2)	O5—Cl—O6	109.20 (12)
O3—C8—H8A	109.5	O7—Cl—O4	109.12 (12)
O3—C8—H8B	109.5	O5—Cl—O4	108.41 (12)
H8A—C8—H8B	109.5	O6—Cl—O4	109.75 (11)

### Hydrogen-bond geometry (Å, °)

**Table:**

*D*—H···*A*	*D*—H	H···*A*	*D*···*A*	*D*—H···*A*
C4—H4···O7^i^	0.93	2.58	3.461 (4)	159
C12—H12···O5^ii^	0.93	2.57	3.499 (3)	177
C15—H15···O5^ii^	0.93	2.59	3.517 (3)	171

Symmetry codes: (i) −*x*+2, −*y*, −*z*+1; (ii) −*x*+1, −*y*+1, −*z*.
